# Outcome comparison between percutaneous cholecystostomy and cholecystectomy: a 10-year population-based analysis

**DOI:** 10.1186/s12893-017-0327-6

**Published:** 2017-12-07

**Authors:** Ping Lu, Chien-Lung Chan, Nan-Ping Yang, Nien-Tzu Chang, Kai-Biao Lin, K. Robert Lai

**Affiliations:** 1 School of Economics and Management, Xiamen University of Technology, Xiamen, 361024 China; 2 Department of Information Management, Yuan Ze University, Taoyuan, 32003 Taiwan; 3 Department of Surgery, Keelung Hospital, Ministry of Health and Welfare, Keelung, 20147 Taiwan; 4 Institute of Public Health, National Yang-Ming University, Taipei, 11221 Taiwan; 5 School of Nursing, College of Medicine, National Taiwan University, Taipei, 10051 Taiwan; 6 Department of Computer Science and Engineering, Yuan Ze University, Taoyuan, 32003 Taiwan; 7 School of Computer & Information Engineering, Xiamen University of Technology, Xiamen, 361024 China; 8 Innovation Center for Big Data and Digital Convergence, Yuan Ze University, Taoyuan, 32003 Taiwan

**Keywords:** Percutaneous cholecystostomy, Cholecystectomy, Acute cholecystitis, Tokyo guidelines

## Abstract

**Background:**

Controversy surrounding the role of percutaneous cholecystostomy (PC) is fed by the absence of large amounts of data concerning its outcomes, and many authors have maintained that there is no evidence to support a recommendation for PC rather than cholecystectomy (CCS) in elderly or critically ill patients with acute cholecystitis (AC).

**Methods:**

We conducted this study by tracking trends in the utilization and outcomes of PC and CCS using longitudinal health research data in Taiwan.

**Results:**

Analyses were conducted on 236,742 patients, 11,184 of whom had undergone PC and 225,558 of whom had undergone CCS. Average annual percentage changes (AAPCs) from 2003 to 2012 increased significantly by 18.34% each year for PC and by 2.82% each year for CCS. The subset analyzes showed that the mortality rates were far higher in patients underwent PC than in patients underwent CCS in all subgroups, which increased from a minimum of 1.45-fold to a maximum of 34.22-fold. The gap of the mortality rates between PC group and CCS group narrowed as the patients aged and with the seriousness of the diseases increased. Most patients with PC or CCS who died in-hospital or within 30 days after discharge were 70 years of age or older, and a large number of them received a CCI score of at least 1. The AAPCs of the overall mortality rates from 2003 to 2012 decreased by 6.78% each year for PC and by 7.33% each year for CCS. PC was related to a higher rate of cholecystitis recurrence and readmission for complications, but a lower rate of in-hospital complications and routine discharge than CCS, and 36.41% of all patients treated with PC underwent subsequent CCS. Additionally, the patients with PC experienced longer hospital stays and generated higher costs than the patients with CCS.

**Conclusion:**

Patients who underwent PC demonstrated poorer prognoses than did patients who underwent CCS. The role of PC in the Tokyo guidelines may be overstated; it is not as safe as the Tokyo guidelines have suggested in moderate-grade cholecystitis cases, and it should be limited to only the elderly and sicker patients.

## Background

While cholecystectomy (CCS) is the standard treatment for acute cholecystitis (AC) [1, 2], this approach poses significant mortality risks from urgent surgery to elderly and critically ill patients [3]. Percutaneous cholecystostomies (PCs), which involve percutaneous, imaging-guided catheter placement in the gallbladder lumen, were first described by Radder in 1980 [4]. This procedure allows for the immediate decompression of an acutely inflamed gallbladder, requires the use of only local anesthesia, thus eliminating the need for surgery, and can serve as either a bridge to surgery or as a definitive treatment designed for unfit patients and for those who refuse to undergo CCS [5–8]. The Tokyo guidelines, first in 2007 and then again in 2013, considered that the use of PC not only as an alternative procedure in critically ill patients but also as a bridge to surgery in patients with moderate-grade cholecystitis [9, 10]. However, PC has not been proven to be an effective alternative to early surgery. Many authors have maintained that there is no evidence to support a recommendation for PC other than CCS in elderly or critically ill patients with AC [11, 12]. Controversy surrounding the role of PC is fed by the absence of reliable data concerning its outcomes. Some studies have reported that the mortality rate is far higher after PC than after CCS, even for critically ill patients [13, 14]. Thus, we could not determine whether the Tokyo guideline recommendations were adequate and current or whether they should be revised due to the lack of large amounts of data.

Although previous epidemiological studies have compared the health outcomes of PC and CCS, most of these studies have been based on hospital-level patient data [15]. Huang et al. [16] conducted a population-based study to assess trends in the incidence of severe gallstone disease in Taiwan among adults aged ≥20 years in 2009, but this study did not refer to PC procedures. To the best of our knowledge, only Anderson et al. [17], in 2013, have conducted a nationwide examination of the outcomes of PC compared to the outcomes of CCS for AC. Therefore, in-depth population-based research and analyses must be conducted to investigate possible roles of PC in the management of AC and other gallbladder diseases. These analyses may lead to treatment suggestions for medical research institutions and surgeons when making decisions concerning the management of patients with AC and the judicious use of PC and CCS.

## Methods

### Study subjects and data source

Taiwan’s National Health Insurance (NHI) program launched in 1995; by 2011, the coverage rate had expanded to 99.9%. All enrollees can access health care services from most hospitals and clinics. The Bureau of NHI established a population-based research database that includes nationwide data that is both high quality and representative. Various data subsets, such as inpatient expenditures, details of prescription orders, and clinic or ambulatory care expenditures, were included in the NHI research database (NHIRD). In this study, the inpatient data and prescription orders by admissions were used for further analysis.

### Data protection and permission

All the subjects’ information was double encrypted to protect the patients’ privacy. All researchers are required to declare and sign a written agreement before using these data subsets. This study was also approved by the research ethics committee of Taoyuan General Hospital (Approval Number: TYGH103015), which has been certified by the Ministry of Health & Welfare of Taiwan, and the research protocol was required to be reviewed by the National Health Research Institutes (Agreement Number: NHIRD-104-081).

### Data definition

To compare the trends and outcomes of PC and CCS in Taiwan, we used International Classification of Diseases, Ninth Revision, Clinical Modification (ICD-9-CM) diagnosis codes. PC was identified as ICD-9-CM procedure code 51.01, and CCS was identified as ICD-9-CM procedure codes 51.2 and 51.21–51.24 [17]. We divided patients who accepted either of the above two operation procedures into PC and CCS patients based on the first operation that they received during their hospital stay. When patients initially underwent PC, they were classified as PC patients. Similarly, patients were classified as CCS patients when they initially underwent CCS. Patients who underwent both PC and CCS operations during the same hospitalization (i.e., PC as a bridge to CCS surgery) were classified as CCS patients. Only patients of no younger than 18 years who had undergone PC or CCS were included. To analyze procedure causes, ACs with a calculus/stone were defined as patients with ICD-9-CM diagnosis codes 574.0, 574.3, and 574.6; ACs without a calculus/stone were defined as patients with ICD-9-CM diagnosis code 575.0; calculus without ACs referred to patients with ICD-9-CM diagnosis codes 574.1, 574.2, 574.4, 574.5, 574.7, 574.8, or 574.9; other disorders of the gallbladder or biliary tract were designated patients with ICD-9-CM diagnosis codes 575 or 576 excluding diagnosis code 575.0; and malignant neoplasms of digestive organs and the peritoneum included patients with ICD-9-CM diagnosis codes 150–159 but omitting diagnosis codes 574, 575, and 576.

### Classification of the low-income population and general population

The enrolled subjects were divided into two groups based on their socioeconomic status, considered a dichotomous variable: the low-income population (LIP) group and the general population (GP) group, based on the criteria of Taiwan’s Social Welfare Act. LIP households were defined as those with monthly incomes of less than the minimum living expense standard of the region of residence. This subgroup was classified into the fifth insured class under the Taiwan NHI database [18]. Family property and the minimum living expense standard were not permitted to exceed 60% of the average monthly disposable income for the corresponding year and for a given region [19]. The GP included all individuals who were not in the LIP.

### Measurement outcomes

#### In-hospital complications

We examined all-cause, nonfatal in-hospital morbidity rates based on ICD-9-CM codes. Complications were grouped into 9 categories (mechanical wound complications, infections, urinary complications, pulmonary complications, systemic complications, complications arising during procedures, specific complications of the gallbladder/digestive system, respiratory complications, others). As the NHIRD DD dataset includes inpatient data only, complications occurring after hospital discharge were not considered in our analysis.

#### Mortality

In-hospital mortality referred to patients who underwent PC or CCS and died during hospitalization. 30-day mortality after discharge referred to patients who died within 30-days after their discharges. Total mortality was calculated by including both of the cases died in the hospital and those who died within 30-days after their discharges.

#### Rate of routine discharge

The NHIRD provides information on patient discharge statuses (1, treated and discharged; 2, continued to hospital; 3, transferred to outpatient treatment; 4, death; 5, discharge against medical advice; 6, referral; 7, change in status; 8, abscond; 9, suicide; 0, other; and A, discharged while dying). Patients were grouped into the following categories: routine discharge (1, 3) and non-routine discharge (0, 2, 4–9, and A).

#### Readmission due to complications

Readmission due to complications was designated when readmission occurred due to the diagnosis of a commonly encountered postoperative complication listed in (Table 5 in Appendix) within 3 months after PC or CCS delivery.

#### Recurrence of Cholecystitis

Cholecystitis recurrence was designated when readmission occurred due to the diagnosis of cholecystitis after PC or CCS surgery.

#### Length of hospital stay (LOS)

The period between admission and discharge was defined as the LOS (measured in days). The LOS was measured as 1 day for patients discharged on the same day that they were admitted to the hospital [20].

#### Hospital costs

Hospital costs were calculated by summing all items enumerated in the hospital discharge summary, including operation-associated costs and ward costs. Operation-associated costs included anesthesia and surgery fees as well as costs of medical supplies used during an operation. Surplus costs were classified as ward costs. Costs are expressed in U.S. dollars (USD). In 2007, one USD dollar was equivalent to approximately 32.64 Taiwan dollars [20].

### Statistical analysis

For the analysis, descriptive statistics for comparing baseline characteristics were determined based on the number of cases, percentages, and 95% confidence intervals (CI) for the estimated rates. Independent t-tests were used to evaluate the significance of differences between two subgroups. Temporal trends were analyzed using joinpoint regression, a statistical method that fits a series of joined straight lines between statistically significant changes in trends (joinpoints). We in turn estimated the change between joinpoints using the National Cancer Institute’s Joinpoint Regression Program Version 4.3.1.0 [21, 22]. Long-term trends over the entire time series were designated average annual percentage changes (AAPCs) and were estimated as the weighted average of short-term annual percentage changes with weights equal to the length of the short-term line segment [23]. Binary logistic regression analysis was used, and odds ratios (ORs) were calculated. All statistical analyses were performed using the Statistical Package for Social Sciences for Windows (SPSS for Windows Version 19.0).

## Results

From 2003 to 2012, a total of 236,742 patients had undergone PC or CCS. Among them, 11,184 patients (4.72%) underwent PC, and the remaining 225,558 patients (95.28%) underwent CCS. The average ages of the patients who had undergone PC and CCS were 70.8 ± 14.6 years and 56.8 ± 16.2 years, respectively. The crude rate of PC was 6.09 per 100,000 per year (95% CI: 4.56–7.62), and the crude rate of CCS was 124.59 per 100,000 per year (95% CI: 117.68–131.51).

**Fig. 1:**
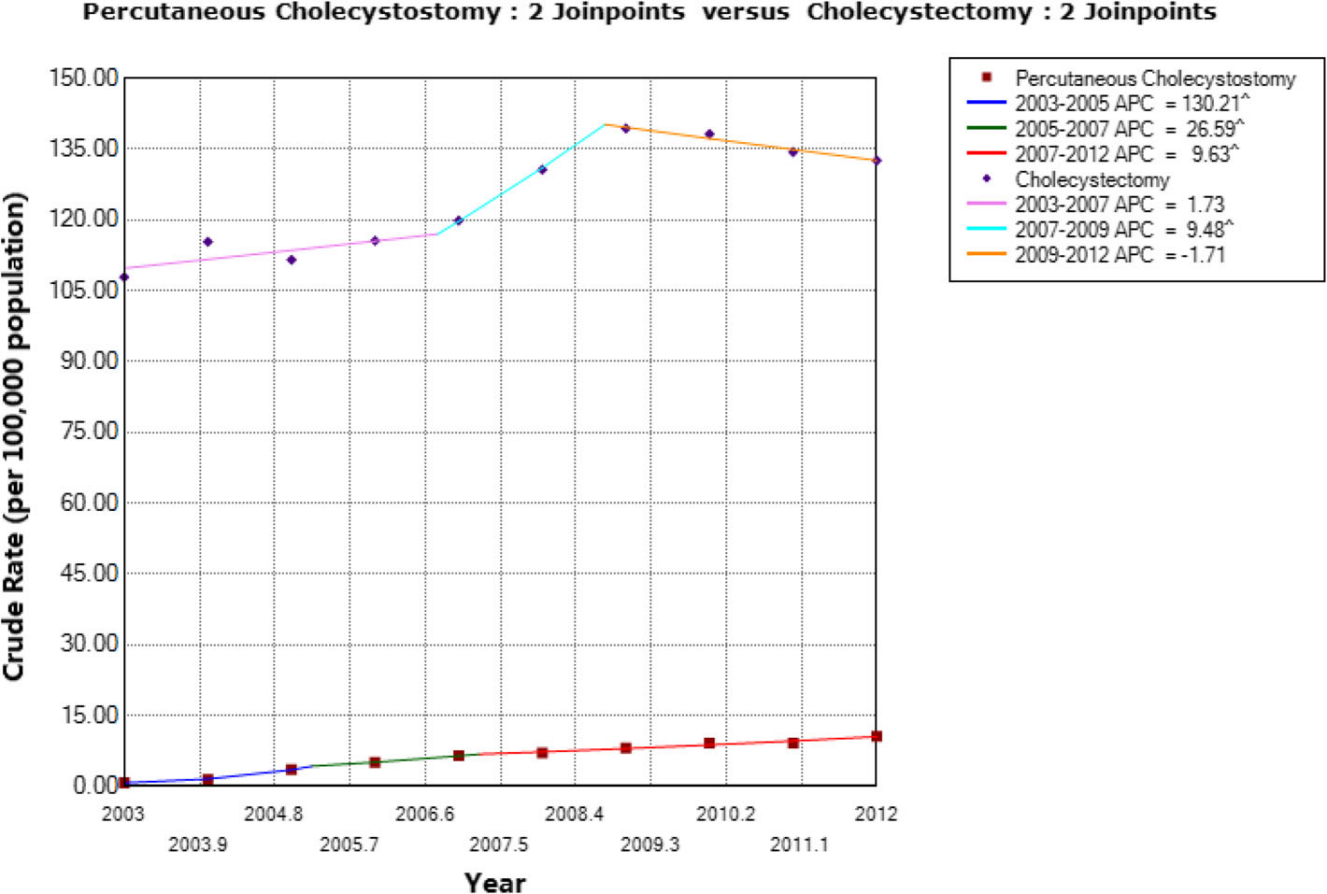
Comparison of the crude rates for patients who underwent percutaneous cholecystostomy and cholecystectomy in Taiwan from 2003 to 2012

**Fig. 2:**
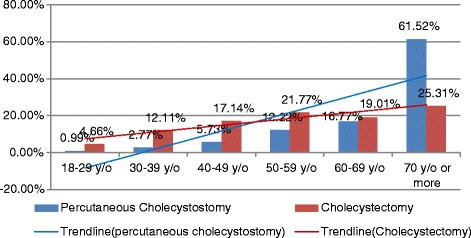
Age-specific proportions of patients who underwent percutaneous cholecystostomy and cholecystectomy in Taiwan from 2003 to 2012

As shown in Fig. 1, long-term trends (AAPCs) between 2003 and 2012 increased significantly by 18.34% per year for patients who underwent PC (from 0.69 to 10.59 per 100,000) and by 2.82% per year for patients who underwent CCS (from 107.88 to 132.67 per 100,000). The short-term trends (APCs) for PC and CCS were also significantly different. The age-specific percentages of patients undergoing PC and CCS are shown in Fig. 2. The proportions of patients who underwent PC showed a very significant growth trend with age; most patients were 70 years of age or older, with this age group accounting for 61.52% of all patients. Although the proportion of CCS for each age group also showed a gradual increasing trend with age, the distribution was relatively uniform for each age group (Fig. 2).

**Fig. 3:**
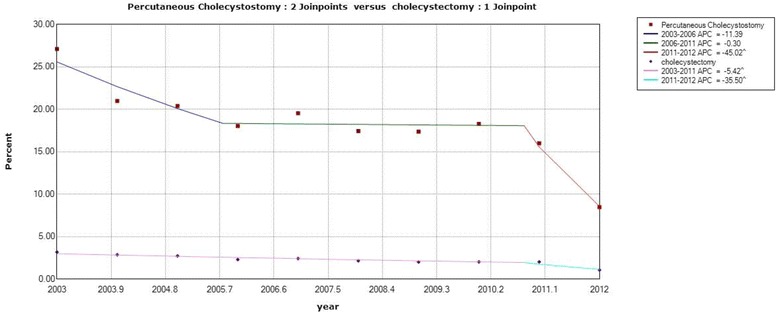
Comparison of mortality rates for patients who underwent percutaneous cholecystostomy and cholecystectomy in Taiwan from 2003 to 2012

The demographic characteristics of the patients undergoing PC and CCS are shown in Table 1. The distributions of PC and CCS varied greatly among the different variables. As shown in Table 2, the patients who underwent PC were associated with higher rates of mortality and readmission due to complications but lower rates of in-hospital complications and routine discharge relative to the patients who underwent CCS. Among the patients who died during hospitalization or within 30 days after CCS, 3103 (62.22%) were male and 1884 (37.78%) were female; on average, the patients were 70.5 ± 14.7 years of age, and 3001 patients (60.18%) had a CCI score of at least 1. Correspondingly, for patients who died during hospitalization or within 30 days after PC, 1102 (60.32%) were male, and 725 (39.68%) were female; on average, the patients were 73.3 ± 14.0 years of age, and 1117 patients (61.14%) had a CCI score of at least 1. As shown in Fig. 3, the AAPCs of total mortality from 2003 to 2012 decreased by 6.78% per year for PC from 27.12% to 8.47% and by 7.33% per year for CCS from 3.16% to 1.06%.

**Table 1:** Demographic characteristics of patients who underwent percutaneous cholecystostomy and cholecystectomy in Taiwan from 2003 to 2012

Variable	Total (*n* = 236,742)		Percutaneous cholecystostomy (*n* = 11,184, 4.72%)		Cholecystectomy (*n* = 225,558, 95.28%)		*P*-value
	n	%	n	%	n	%	
Sex							<0.001
Female	119,108	50.31%	4570	40.86%	114,538	50.78%	
Male	117,634	49.69%	6614	59.14%	111,020	49.22%	
Age stratum							<0.001
18–29 y/o	10,628	4.49%	111	0.99%	10,517	4.66%	
30–39 y/o	27,628	11.67%	310	2.77%	27,318	12.11%	
40–49 y/o	39,299	16.60%	641	5.73%	38,658	17.14%	
50–59 y/o	50,474	21.32%	1367	12.22%	49,107	21.77%	
60–69 y/o	44,744	18.90%	1875	16.77%	42,869	19.01%	
70 y/o or more	63,969	27.02%	6880	61.52%	57,089	25.31%	
Cause of procedure							<0.001
AC with a C/S	68,507	28.94%	4116	36.80%	64,391	28.55%	
AC without a C/S	12,597	5.32%	3424	30.62%	9173	4.07%	
Calculus without AC	116,285	49.12%	1244	11.12%	115,041	51.00%	
ODGBT	15,033	6.35%	1381	12.35%	13,652	6.05%	
MNDOP	18,747	7.92%	354	3.17%	18,393	8.15%	
Others	5573	2.35%	665	5.95%	4908	2.18%	
CCI score							<0.001
0	163,028	68.86%	5494	49.12%	157,534	69.84%	
1	31,985	13.51%	2828	25.29%	29,157	12.93%	
2	23,314	9.85%	1426	12.75%	21,888	9.70%	
≥ 3	18,415	7.78%	1436	12.84%	16,979	7.53%	
Socioeconomic Status							0.007
GP	234,136	98.90%	11,032	98.64%	223,104	98.91%	
LIP	2606	1.10%	152	1.36%	2454	1.09%	
Hospital level							<0.001
Medical center	112,537	47.54%	6265	56.02%	106,272	47.12%	
Regional hospital	105,701	44.65%	4627	41.37%	101,074	44.81%	
District hospital	18,504	7.82%	292	2.61%	18,212	8.07%	

*AC* acute cholecystitis, *C/S* calculus/stone, *ODGBT* other disorders of gallbladder or biliary tract, *MNDOP* malignant neoplasm of digestive organs and the peritoneum, *CCI* Charlson Comorbidity Index, *LIP* low-income population, *GP* general population

**Table 2:** Characteristics of in-hospital complications, in-hospital mortality rates, routine discharge rates, readmissions for complications, and cholecystitis recurrence rates

Variables	All (N, %)	Procedure type (N, %)		*P*-value
		Percutaneous cholecystostomy	Cholecystectomy	
Total mortality ^a^	6814 (2.88%)	1827 (16.34%)	4987 (2.21%)	<0.001
In-hospital mortality	4936 (2.08%)	1334 (11.93%)	3602 (1.60%)	<0.001
30-day mortality after discharge	1878 (0.80%)	493 (4.41%)	1385 (0.61%)	<0.001
In-hospital complications ^b^	9320 (3.94%)	231 (2.07%)	9089 (4.03%)	<0.001
Specific gallbladder/digestive system complications	4758 (2.01%)	84 (0.75%)	4674 (2.07%)	<0.001
Infections	2904 (1.23%)	54 (0.48%)	2850 (1.26%)	<0.001
Mechanical wound complications	1358 (0.57%)	38 (0.34%)	1320 (0.59%)	0.001
Complications during procedures	908 (0.38%)	26 (0.23%)	882 (0.39%)	0.008
Pulmonary complications	363 (0.15%)	53 (0.47%)	310 (0.14%)	<0.001
Systemic complications	160 (0.07%)	4 (0.04%)	156 (0.07%)	0.185
Respiratory	82 (0.03%)	8 (0.07%)	74 (0.03%)	0.032
Urinary complications	22 (0.01%)	0 (0.00%)	22 (0.01%)	0.296
Other	42 (0.02%)	2 (0.02%)	40 (0.02%)	0.991
Rates of routine discharge	229,336 (96.87%)	9092 (81.29%)	220,244 (97.64%)	<0.001
Treatment and discharge	13,208 (5.58%)	739 (6.61%)	12,469 (5.53%)	<0.001
Transferred to outpatient treatment	216,128 (91.29%)	8353 (74.69%)	207,775 (92.12%)	<0.001
Readmission due to complications	3523 (1.49%)	386 (3.45%)	3137 (1.39%)	<0.001
Recurrence of cholecystitis	–	2856 (25.54%)	–	–
Cholecystectomy delivered	–	2419 (21.63%)	–	–
Elective cholecystectomy	–	1652 (14.78%)	–	–

^a^ Total mortality was calculated by including both of the cases died in hospital and those who died within 30-days after their discharges

^b^ Two or more complications occurred for the same patient; thus, the total number of patients with complications was less than the sum of the number of patients with each independent complication

**Fig. 4:**
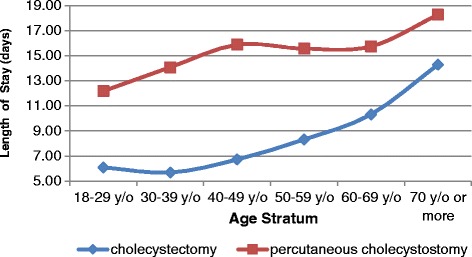
Mean lengths of hospital stay by age group for patients who underwent percutaneous cholecystostomy and cholecystectomy in Taiwan from 2003 to 2012

**Fig. 5:**
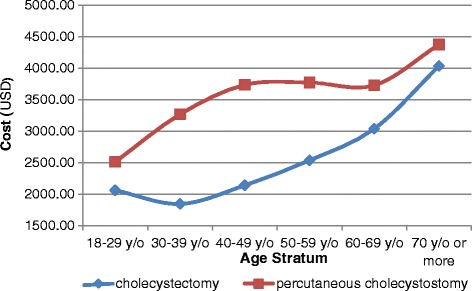
Average hospital costs by age group for patients who underwent percutaneous cholecystostomy and cholecystectomy in Taiwan from 2003 to 2011

**Fig. 6:**
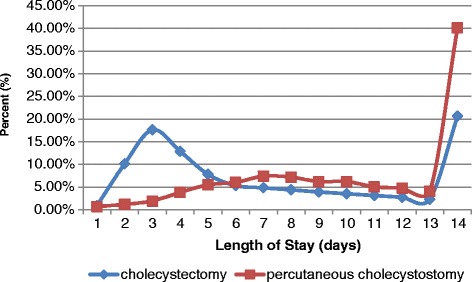
Frequency distributions of hospital length of stay for patients who underwent percutaneous cholecystostomy and cholecystectomy

Table 3 showed that PC was correlated with a significantly longer LOS and a much higher cost than CCS. The age-specific hospitalization times and hospital costs showed that the mean LOS and costs for the patients who underwent PC were higher than those for the patients who underwent CCS across all age groups (Figs. 4 and 5). Compared to the patients who underwent CCS, a significantly higher percentage of the patients who underwent PC had a LOS period longer than 14 days (40.15% vs. 20.63%, *p* < 0.001) (Fig. 6).

**Table 3:** Medical utilization for patients who underwent percutaneous cholecystostomy and cholecystectomy in Taiwan from 2003 to 2012

Procedure type	Summed cases	LOS (days)	Cost (USD)
	2003–2012(%)	Mean ± SE	Mean ± SE
Cholecystectomy	225,558 (95.28%)	9.51 ± 1.06	2836.86 ± 8.59
Percutaneous Cholecystostomy	11,184 (4.72%)	17.20 ± 1.52	4106.71 ± 52.53
*P*-value		<0.001	<0.001

Table 4 shows the relative risk of death among the patients who underwent PC versus the patients who underwent CCS. The in-hospital and 30-day mortality after discharge rates of PC were higher than those of CCS in all subgroups, and the in-hospital and 30-day mortality after discharge rates had similar distributions for each variable.

**Table 4:** Related factors (represented by their odds ratio) of death among patients underwent percutaneous cholecystostomy versus patients underwent cholecystectomy

Stratified variables	In-hospital mortality	30-day mortality after discharge	Total mortality ^a^
	OR (95% CI)	OR (95% CI)	OR (95% CI)
Total	8.35(7.81,8.92) ***	7.31(6.61,8.10) ***	8.64(8.15,9.15) ***
Sex			
Female	11.45(10.31,12.72) ***	8.87(7.54,10.44) ***	11.28(10.29,12.36) ***
Male	6.52(5.99,7.09) ***	6.18(5.43,7.04) ***	6.95(6.46,7.49) ***
Age stratum			
18–29 y/o	12.46(5.22,29.75) ***	9.63(2.22,41.71) **	13.31(6.21,28.53) ***
30–39 y/o	34.22(22.76,51.44) ***	16.23(8.20,32.11) ***	30.57(21.38,43.73) ***
40–49 y/o	20.07(15.15,26.59) ***	15.34(9.84,23.93) ***	20.72(16.22,26.47) ***
50–59 y/o	12.88(10.39,15.96) ***	9.37(6.77,12.96) ***	12.42(10.33,14.94) ***
60–69 y/o	8.34(7.01,9.91) ***	6.62(5.16,8.50) ***	8.25(7.12,9.55) ***
70 y/o or more	3.67(3.38,3.98) ***	3.65(3.21,4.14) ***	3.89(3.62,4.18) ***
Cause of procedures			
AC with a C/S	4.43(3.80,5.17) ***	5.03(4.07,6.21) ***	4.77(4.20,5.42) ***
AC without a C/S	2.62(2.26,3.03) ***	3.59(2.84,4.54) ***	3.02(2.65,3.43) ***
Calculus without AC	13.25(10.89,16.13) ***	7.30(5.20,10.25) ***	11.87(9.97,14.13) ***
ODGBT	12.08(10.18,14.32) ***	10.87(8.44,14.00) ***	13.64(11.74,15.84) ***
MNDOP	10.51(8.24,13.42) ***	6.97(4.87,9.98) ***	11.19(8.95,14.01) ***
Others	4.61(3.83,5.56) ***	3.44(2.36,5.02) ***	5.29(4.42,6.33) ***
CCI score			
0	11.39(10.29,12.62) ***	9.70(8.17,11.51) ***	11.62(10.62,12.72) ***
1	5.19(4.42,6.08) ***	3.88(3.02,5.00) ***	5.01(4.36,5.75) ***
2	6.52(5.56,7.64) ***	5.96(4.75,7.48) ***	7.07(6.16,8.12) ***
≥ 3	4.79(4.16,5.52) ***	4.52(3.73,5.49) ***	5.42(4.79,6.14) ***
Other			
70 y/o or more & AC & CCI ≥ 3	1.45(1.01,2.09) *	2.48(1.51,4.06) ***	1.72(1.07,2.77) ***

*OR* odds ratio, *AC* acute cholecystitis, *C/S* calculus/stone, *ODGBT* other disorders of gallbladder or biliary tract, *MNDOP* malignant neoplasm of digestive organs and the peritoneum, *CCI* Charlson Comorbidity Index

*: *p* < 0.05, **: *p* < 0.01; ***: *p* < 0.001

^a^ Total mortality was calculated by including both of the cases died in hospital and those who died within 30-days after their discharges

## Discussion

The management of gallstone disease in the elderly and critically ill is often more challenging because these patients experience a high incidence of cholelithiasis complications [24], and PC has been described as a safe alternative treatment option for AC in elderly or critically ill patients [1, 3, 13, 17]. Our findings confirm that most patients who undergo PC were elderly or critically ill. For instance, the proportion of PC patients aged 70 years or older was significantly higher than the proportion of CCS patients in this age group (61.52% for PC vs. 25.31% for CCS, *p* < 0.001) (Table 1). Moreover, the proportion of patients with a CCI score of 1 or more was higher among the patients who underwent PC than that among the patients who underwent CCS (50.88% for PC vs. 30.16% for CCS, *p* < 0.001), and the proportion of patients who underwent PC with AC cholelithiasis complications was higher than the proportion of CCS patients (67.42% for PC vs. 32.61% for CCS, *p* < 0.001) (Table 1).

Temporal trends in the crude rate of patients undergoing PC increased significantly by 18.34% per year (AAPCs) from 2003 to 2012, although the levels increased by only 2.82% per year (AAPCs) for CCS over the observed period, and a steady decline of 1.71% per year was found from 2009 to 2012 (Fig. 1). This result shows that PC operations have gained wide acceptance as a form of AC treatment in Taiwan in recent years. This phenomenon is consistent with trends revealed through some previous studies showing that PC use has gradually increased. Lin et al. [8] reported that the rate of PC use markedly increased from 0.5% in 2005 to 12.2% in 2015 and that this procedure has been more commonly applied among the elderly (*p* = 0.009). Duszak Jr. and Behrman [25] reported that annual PC procedures increased by 567% between 1994 and 2009. Smith et al. [26] also reported an increased use of PC for the treatment of AC over a 20-year period at a single institution. The improvement in medical technology and decrease in the mortality levels may be partially attributed to this increased use of PC procedures. Taiwan’s aging population may also have affected the increase in PC use, as PC has been established as a treatment option for AC among elderly and critically ill patients [26, 27], and the number of elderly and critically ill patients may increase as the elderly population ages. Meanwhile, the publication of the Tokyo guidelines in 2007 may also have affected this trend; as shown in Fig. 1, there was a downward trend from 2009 to 2012 for patients undergoing CCS, whereas the crude rate of PC showed a steady upward trend from 2003 to 2012.

The controversy over the role of percutaneous gallbladder drainage is based on the opinion expressed by some authors that mortality is far higher after PC than CCS, even for critically ill patients [4, 5, 13]. Winbladh et al. [13] reviewed 53 studies and found that the 30-day and in-hospital mortality rates after PC were high (15.4%), whereas the rates for those treated with CCS were low (4.5%). Aljundi et al. [5] reported a 30-day mortality rate of 16.7% for patients after PC. Campanile et al. [28] conducted a survey of the literature and showed that the in-hospital mortality rate for cholecystostomy varied between 4 and 50% and that the associated morbidity rate varied between 8.2 and 62%. It is unclear whether this difference in mortality is attributable to the fact that patients who underwent PC are at a higher risk of mortality than patients who underwent CCS [13]. In the present study, we also found that the in-hospital and 30-day mortality after discharge rates were significantly higher among patients who underwent PC than among patients who underwent CCS (Table 2). This result is consistent with the rates reported in some existing studies, as mentioned above. However, because the general conditions are far worse in the average PC patient than in the average CCS patient, we cannot conclude that mortality is higher for PC than for CCS from simple comparisons. Instead, in-depth subset analyses must be conducted to justify this finding. Therefore, we compared the mortality rates between patients who underwent PC and CCS stratified by sex, age, cause of procedure and CCI score group.

The results are shown in Table 4. The mortality rates were far higher among patients who underwent PC than among patients who underwent CCS in all subgroups, which increased from a minimum of 1.45-fold to a maximum of 34.22-fold. Based on the subset analyses, we conclude that PC is not as safe as the Tokyo guidelines suggested in moderate-grade cholecystitis cases and actually its mortality rate is higher than that of CCS, even in the worst scenario (elderly patients with AC and a CCI score of 3 or more) and that the relative risk of death for PC is 1.45-fold higher than that for CCS. We also found that the gap of the in-hospital and 30-day mortality after discharge rates narrowed as the patients aged and with the seriousness of the diseases. In other words, the relative risk of death is higher in less compromised patients but lower in the most serious patients. This important aspect means that PC should be limited only to elderly and sicker patients because in all other patients, the risk seems to be excessive. Additionally, we found that most patients with PC or CCS who died in the hospital or within 30 days after an operation were 70 years of age or older (73.3 ± 14.0 years old after PC and 70.5 ± 14.7 years old after CCS). Moreover, a large number of these patients generated a CCI score of 1 or more (61.14% after PC and 60.18% after CCS), which indicates that being elderly and critically ill may cause patients to be more likely to die during hospitalization or within 30 days after discharge for both types of operations.

As shown in Fig. 3, long-term trends of overall mortality from 2003 to 2012 for both operations declined significantly. For PC, the overall mortality rate decreased from 27.12% in 2003 to 8.47% in 2012. These improved mortality rates for recent years align with the results of some previous studies. Winbladh et al. [13] summarized 52 papers and stated that the total mortality rate listed in earlier papers was 22.1% (20 papers published before 1996), while this rate was reported at 13.3% in more recent studies (32 papers published after 1995), revealing a significantly decreasing trend. Smith et al. [26] reported that the 30-day mortality rate decreased from 36 to 12% among patients undergoing PC from 1998 to 2009, and these authors suggested that patient selection procedures may play a major role in these reduced mortality rates. In addition, we found that the proportion of males was significantly higher than that of females among patients who died during hospitalization for both operation types (60.32% were male for PC, 62.22% were male for CCS, respectively), although the total number of patients was closely balanced between males and females (50.31% for females vs. 49.69% for males, *p* < 0.001), indicating that males may be more vulnerable than females.

According to our study, PC was associated with a lower rate of in-hospital complications than CCS (2.07% vs. 4.03%, *p* < 0.001). However, of the 36.41% of patients treated with PC who underwent subsequent CCS procedures, 14.78% of them underwent elective CCS, and 21.63% of patients developed recurrent cholecystitis. Winbladh et al. [13] also reported that more than 40% of all patients eventually undergo CCS surgery, which is consistent with our results. In addition, compared to CCS cases, PC cases were found to be associated with a lower rate of routine discharge (81.29% after PC and 97.64% after CCS), reflecting the poor prognosis of patients with PC relative to that of patients with CCS (Table 2). The costs and LOS values for patients undergoing PC were significantly higher than those for patients undergoing CCS. If we do not consider the age difference for the overall subpopulation, as shown in Figs. 4 and 5, the costs and LOS values for the PC patients are higher than those for the CCS patients for each age stratum. Anderson et al. [17] also found higher mean LOS values and costs for patients undergoing PC (for both acute calculus and acalculus cholecystitis) than for patients receiving CCS even after adjusting for age, race, sex, CCI, teaching hospital status, and year variables.

The present study has some limitations. First, we could not obtain complete details on the general conditions for all patients due to the limitations of the data, which made it difficult to accurately determine the illness severity and thus made it difficult to group the patients in homogeneous PC and CCS groups. However, to the best of our knowledge, the results concerning the relative risk of mortality are stratified by the patients’ general conditions, to the greatest extent possible, including sex, age, cause of procedure and CCI score groups. Second, some patients who underwent PC could not have tolerated any surgery, but we cannot identify these patients, which is an important selection bias in our study. Third, similar to in other administrative and database-based studies, detailed clinical data and examination information could not be obtained in this study. As we could not review the individual medical records to ensure that the records were coded precisely, deviations may exist between the codes and the actual severity of disease conditions. Finally, data on postoperative conditions were also not included. Even so, this national population-based claims database can be recognized as reliable because it has been adopted in many research fields and numerous high-impact publications.

## Conclusions

In conclusion, the present study found that patients after PC had some poor prognoses compared with patients after CCS, such as a higher rate of mortality and cholecystitis recurrence, but a lower rate of routine discharge. Furthermore, the subset analyses demonstrated that the mortality rates were far higher in the patients who underwent PC than in the patients who underwent CCS in all subgroups, even in the worst scenario (elderly patients with AC and a CCI score of 3 or more), but the gap of the mortality rates between PC group and CCS group narrowed as the patients aged and with the seriousness of the diseases increased. The Tokyo guidelines considered the use of PC mandatory for “severe” cases and strongly suggested that this procedure be used even in most moderate-grade cholecystitis cases; however, the present study determined that the role of PC in the Tokyo guidelines may be overstated. It is not as safe as the Tokyo guidelines suggested in moderate-grade cholecystitis cases, and it should be limited only to elderly and sicker patients. But still, as medical technology has improved, the mortality rates of PC have decreased, and the aging population has increased, we suggest strengthening and paying more attention to the use of PC technology in elderly and seriously ill patients.

## Appendix

**Table 5:** ICD-9 codes for postoperative in-hospital complications

Complications	ICD-9 Codes
Mechanical wound complications	998.12, 998.13, 998.3, 998.6, and 998.83
Infections	998.5, 998.51, and 998.59
Urinary complications	997.5
Pulmonary complications	512.1, 518.4, 518.5, and 997.3
Systemic complications	998.0 and 998.89
Complications during procedure	998.11, 998.2, and 998.4
Specific complications of the gallbladder/digestive system	997.4, 576.0, 998.6, 576.4, 575.5, 51.36, 51.37, 51.39, 51.71, 51.72, and 51.79
Respiratory	997.3, 997.31, 997.39
Other	998.4, 998.7, 998.8, 998.89, 998.9

## Abbreviations

AAPC: Average annual percentage change
AC: Acute cholecystitis
APC: Short-term trend
CCI: Charlson Comorbidity Index
CCS: Cholecystectomy
CI: Confidence interval
GP: General population
LIP: Low-income population
LOS: Length of hospital stays
NHI: National Health Insurance
NHIRD: National Health Insurance Research Database
PC: Percutaneous cholecystostomy
